# Supplementary material for: The ATPases of cohesin interface with regulators to modulate cohesin-mediated DNA tethering
Source: eLife. 2015 Nov 19;4:e11315. doi: 10.7554/eLife.11315 (PMC4709263; doi:10.7554/eLife.11315)
Supplement: Supplementary file 1. — DOI: http://dx.doi.org/10.7554/eLife.11315.033 [file elife-11315-supp1.docx]

**Supplemental Table 1. Yeast strain table**

***S. pombe* strains:**

**Name Genotype**

**4483** *h-* *pREP1-Mis4-HA-3C-ProteinA(LEU2)*

*pREP2-Ssl3 (URA4) leu1-32 ura4-D18*

***S. cerevisiae* strains:**

(All *S. cerevisiae* strains are A364A background)

**Name Genotype**

**4443** *Mata pGAL1-PSM3-3xPK-7xH pGAL10-PSM1::URA3 psi+*

*pGAL1-RAD21-HA-3C-ProteinA pGAL10-7xH-PSC3::LEU2 ade2-1 can1-100 leu2-3,112 his3-11,15 ura3 trp1 GAL pep4∆::HIS3*

**VG3460-2A** *Mata LacO(DK)-NAT:10kb-CEN4 trp1-1 leu2-3,112*

*pHIS3-GFPLacI-HIS3:his3-11,15 ura3-52 bar1 GAL+*

**VG3502-2A** VG3460-2A except *rad61∆::HPH ctf7∆::G418*

**VG3547-3B** VG3502-2A except *smc3-D1189H*

**VG3574-5A** VG3502-2A except *smc1-D1164E*

**VG3576-1C** VG3502-2A except *smc1-Y1128C*

**VG3349-1B** *Mata LacO(DK)-NAT::lys4 trp1-1 bar1*

*pHIS3-GFPLacI-HIS3:his3-11,15 leu2-3,112 ura3-52 GAL+*

**VG3503-4A** VG3349-1B except *rad61∆::HPH ctf7∆::G418*

**VG3575-2C** VG3503-4A except *smc1-D1164E*

**VG3576-1C** VG3503-4A except *smc1-Y1128C*

**VG3598-8A** VG3460-2A except *smc1-D1164E-LEU2:leu2-3,112 smc1∆::HPH trp1-1*

**VG3581-8B** VG3349-1B except *smc1-D1164E-LEU2:leu2-3,112 smc1∆::HPH trp1-1*

**VG3623-2A** VG3460-2A except *SMC1-LEU2:leu2-3,112 smc1∆::HPH rad61∆::G418*

**VG3625-2C** VG3460-2A except *smc1-D1164E-LEU2:leu2-3,112 smc1∆::HPH rad61∆::G418*

**VG3601-8B** VG3349-1B except *rad61∆::G418 SMC1-LEU2:leu2-3,112 smc1∆::HPH*

**VG3603-3D** VG3349-1B except *rad61∆::G418 smc1-D1164E-LEU2:leu2-3,112 smc1∆::HPH*

**VG3779-1E** VG3460-2A except *smc1-D1164E-LEU2:leu2-3,112 eco1∆::G418 smc1∆::HPH*

**VG3646-1A** VG3349-1B except *SMC1-LEU2:leu2-3,112 smc1∆::HPH*

*ECO1-3V5-AID2-G418 TIR1-CaTRP1*

**VG3661-1C** VG3349-1B except *smc1-D1164E-LEU2:leu2-3,112 smc1∆::HPH*

*ECO1-3V5-AID2-G418 TIR1-Ca*

**VG3568-8A** VG3349-1B except *smc1∆::HPH + w/pTH2 (SMC1 URA3 CEN)*

**VG3464-16C** VG3349-1B except *smc3∆::HPH + w/(SMC3 CEN URA3)*

**VG3711-5D** VG3349-1B except *SMC1-D604-3V5-AID TIR1-CaTRP1*

**VG3764-3A** VG3711-5D except *SMC1-R373-3FLAG-LEU2:leu2-3,112*

**VG3765-3D** VG3711-5D except *SMC1-R373-3FLAG-D1164E-LEU2:leu2-3,112*

**VG3651-3D** VG3349-1B except *SMC3-N607-3V5-AID TIR1-CaTRP1*

**VG3771-10C** VG3651-3D except *SMC3-N607-6HA-URA3:ura3-52*

**VG3773-16D** VG3651-3D except *SMC3-N607-6HA-D1161E-URA3:ura3-52*

**VG3810-2B** VG3460-2A except *SMC1-D604-3V5-AID TIR1-CaTRP1*

**VG3794-2E** VG3810-2B except *SMC1-R373-3FLAG-LEU2:leu2-3,112*

**VG3795-2G** VG3810-2B except *SMC1-R373-D1164E-3FLAG-LEU2:leu2-3,112*

**VG3811-3D** VG3460-2A except *SMC3-N607-3V5-AID TIR1-CaTRP1*

**VG3797-1A** VG3811-3D except *SMC3-N607-6HA-URA3:ura3-52*

**VG3799-3C** VG3811-3D except *SMC3-N607-6HA-D1161E-URA3:ura3-52*

**VG3766-3C** VG3711-5D except *SMC1-R373-3FLAG-D1164A-LEU2:leu2-3,112*

**VG3772-13A** VG3651-3D except *SMC3-N607-6HA-D1161A-URA3:ura3-52*

**VG3796-1F** VG3810-2B except *SMC1-R373-D1164A-3FLAG-LEU2:leu2-3,112*

**VG3798-2B** VG3811-3D except *SMC3-N607-6HA-D1161A-URA3:ura3-52*

**GC72-4A** 4443 except *GAL-PSM1 D1167A-PSM3 (URA3) ura3*

**GC71-4A** 4443 except *GAL-PSM1 D1167E-PSM3 (URA3) ura3*

**GC70-2A** 4443 except *GAL-PSM1-PSM3 D1132A (URA3) ura3*

**GC64-4A** 4443 except *GAL-PSM1-PSM3 D1132E(URA3) ura3*

**GC54-319** 4443 except *GAL-PSM1-PSM3 K38I (URA3) ura3*
